# The Effect of Hydrothermal Treatment on Metabolite Composition of Hass Avocados Stored in a Controlled Atmosphere

**DOI:** 10.3390/plants10112427

**Published:** 2021-11-10

**Authors:** Rosana Chirinos, David Campos, Sofía Martínez, Sílfida Llanos, Indira Betalleluz-Pallardel, Diego García-Ríos, Romina Pedreschi

**Affiliations:** 1 Instituto de Biotecnología, Universidad Nacional Agraria La Molina, Av. La Molina s/n, La Molina, Lima 12056, Peru; chiri@lamolina.edu.pe (R.C.); 20120453@lamolina.edu.pe (S.M.); 20130806@lamolina.edu.pe (S.L.); ibp@lamolina.edu.pe (I.B.-P.); hect.gar93@gmail.com (D.G.-R.); 2 Escuela de Agronomía, Pontificia Universidad Católica de Valparaíso, Calle San Francisco s/n, La Palma, Quillota 2260000, Chile

**Keywords:** avocado, postharvest treatment, primary and secondary metabolites, harvest stages, edible ripeness

## Abstract

Avocado cv. Hass consumption has expanded worldwide given its nutritional, sensory, and functional attributes. In this work, avocado fruit from two harvests was subjected to hydrothermal treatment (38 °C for 1 h) or left untreated (control) and then stored for 30 and 50 days in a controlled atmosphere (4 kPa O_2_ and 6 kPa CO_2_ at 7 °C) (HTCA and CA, respectively) with subsequent ripening at ~20 °C. The fruit was evaluated for primary and secondary metabolites at harvest, after storage, and after reaching edible ripeness. A decrease from harvest to edible ripeness in mannoheptulose and perseitol was observed while β-sitosterol, hydrophilic and lipophilic antioxidant activity (H-AOX, L-AOX), abscisic acid, and total phenolics (composed of *p*-coumaric and caffeic acids such as aglycones or their derivatives) increased. HTCA fruit at edible ripeness displayed higher contents of mannoheptulose, perseitol, β-sitosterol, L-AOX, caffeic acid, and *p*-coumaric acid derivatives, while CA fruit presented higher contents of α-tocopherol, H-AOX, and syringic acid glycoside for both harvests and storage times. The results indicate that a hydrothermal treatment prior to CA enables fruit of high nutritional value characterized by enhanced content of phenolic compounds at edible ripeness to reach distant markets.

## 1. Introduction

Avocado (*Persea americana* Mill.) is a fruit widely appreciated and demanded throughout the world. It is considered an excellent source of nutrients and phytochemicals with remarkable bioactive properties [1]. The fruit stands out for its high oil content and being rich in unsaturated fatty acids, especially oleic acid [2,3]. In addition, avocado fruit mesocarp is an extremely rich source of bioactive phytochemicals, including C_7_ sugars (e.g., mannoheptulose and perseitol), vitamin E, carotenoids, sterols, and others with antioxidant and radical scavenging activities [4,5]. Recent studies have shown that avocado may improve hypercholesterolemia and may be useful in the treatment of hypertension and type 2 diabetes mellitus, playing an important role in cardiovascular health [6]. 

The cultivar ‘Hass’ represents 90% of the volume commercialized in the world. Among the main producing and exporting countries are Mexico, Peru, Chile, and Spain [7]. These countries have very different climatic conditions and management systems, which leads to great variation in the chemical composition of the commercialized fruit and in their postharvest performance [8]. In addition, the harvest stage based on dry matter or oil content (early, middle, and late) affects its composition in terms of primary and secondary metabolites [5,9]. The export of avocados from the Southern Hemisphere of the American continent, such as Peru and Chile, to distant markets demands maritime transport times of up to 50 days. Thus, postharvest technology and treatments are imperative to ensure the provision of a high internal and external quality, besides satisfying the demands of the consumer related to nutrition and health attributes, which is directly linked to the presence of primary and secondary metabolites of the mesocarp. 

Different postharvest technologies are used and proposed to enhance avocado quality, prolong its shelf-life, modulate the ripening process for consumption, and for phytosanitary treatment. These technologies or postharvest treatments include but are not limited to cold storage, controlled atmosphere, modified atmosphere packaging, and heat treatments [10,11,12,13]. The application of the different treatments, separately or in combination, depends on the desired postharvest conditions (storage time and transport destination). These postharvest technologies and treatments will have an impact on the quality attributes of the fruit, which are closely related to its composition at the level of physicochemical characteristics and the pool of metabolites it contains [12]. Recent studies have reported the evolution of fatty acids and secondary metabolites (e.g., carotenoids, tocopherols, phytosterols, and phenolic compounds) of avocados by simulating only regular air cold storage for a prolonged time [1,5,14]. These studies reported that in general major phytochemicals are maintained during prolonged cold storage while some are triggered or enhanced at edible ripeness. Postharvest heat treatments have previously been applied for insect disinfestation, disease control, and to modify fruit responses to cold stress to maintain fruit quality [10,15] and prior to controlled atmosphere storage to successfully synchronize ripening [12,16]. Previous work has not focused on a thorough evaluation of the impact of postharvest technologies used to export Hass avocado to distant markets (e.g., to European and Asian markets with travel times of 30 and 50 days, respectively) on the main primary and secondary metabolites from harvest to edible ripeness. Thus, in this research, we aim to evaluate the effect of hydrothermal treatment in avocado cv. Hass prior to prolonged controlled atmosphere storage (HTCA) for different simulated travel times (30 and 50 days) and two harvest fruit stages (early and middle) on the evolution of the primary and secondary metabolites and antioxidant activity from harvest, after prolonged storage/transport, and at edible ripeness.

## 2. Results

### 2.1. Differences at Harvest in Primary and Secondary Metabolites and Antioxidant Activity between Early and Middle Harvest Fruit

Of the total characteristics evaluated (22) in avocados at harvest (Initial, 0 day), ten showed significant differences (*p* < 0.05) between early (SI) and middle (SII) harvest. These were dry matter (DM), oil, fructose, mannoheptulose, sucrose, glucose, quinic acid, hydroxybenzoic acid glycoside (HBAG), and abscicic acid (ABA) (Table 1 and Table 2). The contents of DM, oil, ABA, and sucrose significantly increased (14.5, 27.8, 32.4, and 58.1%, respectively) from early to middle harvest, while mannoheptulose, glucose and fructose decreased (38.1, 100 and 68.5%, respectively) and perseitol displayed a nonsignificant decreasing trend. Malic acid followed by quinic acid were the main acids detected in the avocado mesocarp and only quinic acid showed a significant decrease (68.2%) as the harvest progressed. Only one phenolic compound, HBAG, was found in recently harvested avocados and showed a significant reduction (~50%), from early to middle harvest. 

The evaluated lipophilic compounds at harvest (fatty acids, tocopherols, and phytosterols, Table 2) did not present significant changes between harvests (early and middle, respectively). The fatty acids present in Hass avocados in order of highest to lowest percentage of participation were: oleic > palmitic > palmitoleic–linoleic > α-linolenic. α- and β-tocopherols were also detected in the fruit, highlighting the content of α-tocopherol, whereas β-sitosterol corresponded to the phytosterol with the highest content followed by campesterol. 

Finally, the in vitro hydrophilic (H-AOX) and lipophilic (L-AOX) antioxidant activities determined with the ABTS radical scavenging assay (Table 2) showed five to six-fold higher values for H-AOX than L-AOX but between harvests (early and middle) no significant changes were observed for antioxidant activities from harvest to edible ripeness.

### 2.2. Changes in Primary and Secondary Metabolites and Antioxidant Activity after Postharvest Treatments and at Edible Ripeness for Early and Middle Harvest Fruit

After postharvest storage (30 and 50 days), for CA (control) or HTCA (hydrothermal treatment followed by controlled atmosphere) and at edible ripeness (CASL or HTCASL), different trends in sugars and organic acids were observed (Table 1). Thus, perseitol and mannoheptulose decreased progressively and significantly until reaching edible ripeness. Considering the evolution of both sugars from the beginning of harvest and grouping the CA and HTCA and days of storage (30 and 50 days), a greater decrease in mannoheptulose content was observed compared to perseitol, with this decrease being much greater for the early harvest (~95.1–97.3% for mannoheptulose and ~83.2–89.8% for perseitol) than the middle harvest (86.4–95.5% for mannoheptulose and 66.1–82.0% for perseitol). Additionally, at edible ripeness, a greater decrease in mannoheptulose and perseitol was observed for CA (95.1–95.5% and 82.0–89.8%, respectively) than HTCA (86.4–97.3% and 66.18–83.2%, respectively) for both harvests and days of storage (Table 1). Regarding other sugars, it was observed that at edible ripeness, sucrose content in CA stored avocados was higher (especially for middle harvest fruit) than HTCA treated fruit, which remained constant with respect to the initial values (0 day). When establishing the same comparison with glucose and fructose, no clear trend was observed; however, when comparing the control and hydrothermal fruit samples at edible ripeness, HTCA stored avocados presented a slightly higher content of both sugars than CA treated fruit. For the organic acids, malic and quinic, the former was present at levels 3.4 and 10.1-fold higher than those of quinic acid. Neither acid showed significant changes in their concentrations at the different storage times under CA and HTCA, with only the single exception of quinic acid of middle harvest under CA, where a significant increase was evidenced, as well as at edible ripeness (Table 1).

From harvest to edible ripeness of avocados, different trends were observed for lipophilic metabolites (Table 2). The composition of the avocado fatty acids was not influenced by the hydrothermal treatment or the storage time (30 and 50 days). The contents of α- and β-tocopherols remained at similar values after 30 and 50 days of postharvest storage either in CA or HTCA, except for a notable decrease observed for α-tocopherol for middle harvest fruit subjected to HTCA (30 and 50 days). At edible ripeness (Table 2), CA stored fruit displayed a significant increase in α-tocopherol in both harvests (20.6–34.2%). β-tocopherol at edible ripeness did not significantly change with respect to the initial harvest stage (0 day). Additionally, for fruit hydrothermally treated, β-sitosterol increased for middle harvest HTCA fruit, and at edible ripeness (HTCASL) for both harvest stages this compound increased (range between 15.3 and 32.2%) (Table 2), while no changes in campesterol contents were observed. Campesterol remained constant during the whole evaluation period (from harvest to edible ripeness). 

The phytohormone ABA for early harvest fruit significantly increased in control and hydrothermally treated fruit compared to the initial value (0 day), with this being more pronounced for CA than HTCA. Instead, at edible ripeness, ABA content increased up to 10-fold compared to harvest for CA and HTCA stored fruit with no significant differences between the two treatments (Table 2). 

The phenolic compounds identified at harvest and after postharvest treatment followed by CA storage (CA and HTCA) and at edible ripeness (CASL and HTCASL) are presented in Table 3. The compounds belonged to two main families of phenolic compounds: benzoic acids (hydroxybenzoic and syringic acids derivatives) and cinnamic acids (caffeic, *p*-coumaric and trans-ferulic acid derivatives, and *p*-coumaric acid), present in the form of simple aglycone or with glycosylations or acylations. Hydroxybenzoic acid glycoside (HBAG, peak 5), was the only phenolic compound found at harvest in avocado mesocarp (early and middle harvest fruit), and was also the only compound determined after CA storage (30 and 50 days) for both postharvest treatments (control and hydrothermally treated). Values of HBAG at edible ripeness were present in higher contents in CA than HTCA. At edible ripeness, a total of 10 and 13 new phenolic compounds were found for the CA and HTCA treatments, respectively, with the family of cinnamic acids being the most representative one. Of the phenolics found in this study at edible ripeness, the *p*-coumaric acid derivative (*p*-CAD2, peak 12) was the most representative, especially for the middle harvest fruit for both CA and HTCA. Other important compounds in considerable quantities were syringic acid glycoside (SAG), *p*-coumaric acid glycoside (*p*-CAG), and *p*-coumaric acid (*p*-CA) (peaks 2, 6 and 10), also present in higher quantities in middle than early harvest fruit. Total phenolic compounds significantly increased from harvest to edible ripeness from 2.5 to 8.1 fold for CA and from 1.2 to 9.1 fold for HTCA, with higher increases for middle harvest fruit.

A continuous increase in H-AOX was observed from harvest, after avocado storage for CA and HTCA, reaching the maximum values at edible ripeness (Table 2). L-AOX displayed different trends depending on the postharvest hydrothermal treatment. In general, it remained constant from harvest until edible ripeness for CA while for HTCA the values significantly increased at edible ripeness (Table 2). Finally, the length of storage (30 and 50 days) did not affect the contents of primary and secondary metabolites evaluated as well as the in vitro antioxidant activity for CA and HTCA fruit.

### 2.3. Multivariate Analysis of Primary and Secondary Metabolites and Antioxidant Activities According to Harvest Stages and Postharvest Treatments

**Figure 1:**
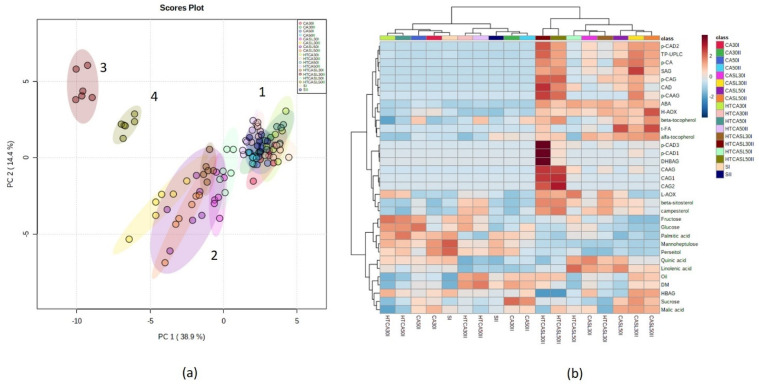
Principal component analysis of the complete dataset, including early and middle harvest fruit. (**a**) Score plot and (**b**) heat map based on hierarchical cluster analysis based on Euclidean distance and Ward algorithm with the 33 significant metabolites revealed by ANOVA followed by the Tukey test (*p* < 0.05). Samples correspond to different treatments, harvest stages, storage times, and corresponding edible ripeness stages. Samples corresponding to the different conditions are on the horizontal axis, and the abundance of the different metabolites and analysis are represented on the vertical axis. SI = Initial day early harvest fruit; SII = Initial day middle harvest fruit; CA30I and CA50I = controlled atmosphere for 30 and 50 days early harvest; CA30II and CA50II = controlled atmosphere for 30 and 50 days middle harvest; CASL30I and CASL50I = controlled atmosphere for 30 and 50 days early harvest after shelf-life period; CASL30II and CASL50II = controlled atmosphere for 30 and 50 days middle harvest after shelf-life period; HTCA30I and HTCA50I = hydrothermal treatment for 30 and 50 days early harvest; HTCA30II and HTCA50II = hydrothermal treatment for 30 and 50 days middle harvest; HTCASL30I and HTCASL50I = hydrothermal treatment for 30 and 50 days early harvest after shelf-life period; HTCASL30II and HTCASL50II = hydrothermal treatment for 30 and 50 days middle harvest after shelf-life period. Average of 6 replicates is displayed. DM = dry matter, ABA = abscisic acid, H-AOX = hydrophilic antioxidant activity, L-AOX = lipophilic antioxidant activity, DHBAG = dihydroxybenzoic acid glycoside, SAG = syringic acid glycoside, CAG1 = caffeic acid glycoside 1, CAG2 = caffeic acid glycoside 2, HBAG = hydroxybenzoic acid glycoside, *p*-CAG = *p*-coumaric acid glycoside, CAD = caffeic acid derivative, *p*-CAD1 = *p*-coumaric acid derivative, CAAG = caffeic acid acetilglycoside, *p*-CA = *p*-coumaric acid, *p*-CAAG = *p*-coumaric acid acetylglycoside, *p*-CAD2 = *p*-coumaric acid derivative, *t*-FA = trans-ferulic acid, *p*-CAD3 = *p*-coumaric acid derivative.

Principal component analysis (PCA) analysis considering all data together (CA and HTCA, harvest stages, storage time, and corresponding edible ripeness stages) revealed four clusters (numbers 1 to 4) and 53.3% of total variance could be explained with the first two components. Samples at harvest, regardless of harvest (early and middle) and treatment (CA and HTCA) and storage time (30 and 50 days), clustered together (Figure 1, cluster 1), which indicates that neither primary nor secondary metabolites changed much during simulation of travel to distant markets and were correlated with higher contents of sugars (mannoheptulose, perseitol, fructose, and glucose) and palmitic acid. Cluster 2 clearly grouped the samples at edible ripeness, including samples of CA of early and middle harvests (CASL30I and 50I and CASL30II and 50II) and hydrothermally treated (early harvest stage only, HTCASL30I, and 50I). These samples presented lower contents of sugars and palmitic acid but higher amounts of quinic acid and H-AOX, t-FA, and α-tocopherol.

Cluster 3 and 4 grouped samples corresponding to the hydrothermal treatment at edible ripeness of middle harvest fruit after 30 and 50 days (HTCASL30II and 50II), respectively, and was positively correlated with different phenolics and cinnamic acids, such as *p*-CAD3, *p*-CAD1, CAAG, CAG1, and CAG2. A clear higher content of TP-UPLC, ABA, and cinnamic acids (*p*-CAD2, *p*-CAG, *p*-CA, and SAG) was observed in all samples at edible ripeness (Figure 1). A biplot is presented in Figure S1.

**Figure 2:**
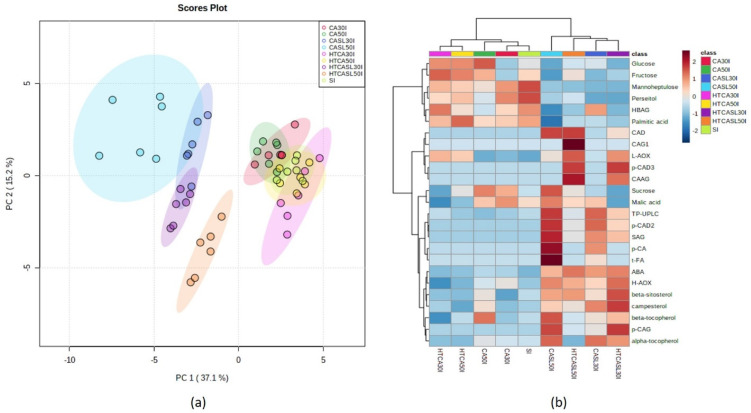
Principal component analysis of early harvest dataset. (a) Score plot and (b) heat map based on hierarchical cluster analysis based on Euclidean distance and Ward algorithm with the 25 significant metabolites revealed by ANOVA followed by the Tukey test (*p* < 0.05). Samples correspond to different treatments, storage times, and corresponding edible ripeness stages. Samples corresponding to the different conditions are on the horizontal axis, and the abundance of the different metabolites and analysis are represented on the vertical axis. SI = Initial day early harvest; CA30I and CA50I = controlled atmosphere for 30 and 50 days early harvest; CASL30I and CASL50I = controlled atmosphere for 30 and 50 days early harvest after shelf-life period; HTCA30I and HTCA50I = hydrothermal treatment for 30 and 50 days early harvest; HTCASL30I and HTCASL50I = hydrothermal treatment for 30 and 50 days early harvest after shelf-life period. The average of the 6 replicates is displayed. DM = dry matter, ABA = abscisic acid, H-AOX = hydrophilic antioxidant activity, L-AOX = lipophilic antioxidant activity, DHBAG = dihydroxybenzoic acid glycoside, SAG = syringic acid glycoside, CAG1 = caffeic acid glycoside 1, CAG2 = caffeic acid glycoside 2, HBAG = hydroxybenzoic acid glycoside, *p*-CAG = *p*-Coumaric acid glycoside, CAD = caffeic acid derivative, *p*-CAD1 = *p*-coumaric acid derivative, CAAG = caffeic acid acetilglycoside, *p*-CA = *p*-coumaric acid, *p*-CAAG = *p*-coumaric acid acetylglycoside, *p*-CAD2 = *p*-coumaric acid derivative, *t*-FA = trans-ferulic acid, *p*-CAD3 = *p*-coumaric acid derivative.

The same PCA and hierarchical cluster analysis was performed on data from each harvest (early and middle separately). Early harvest fruit (I) dataset PCA analysis revealed three marked clusters. Samples were mainly differentiated by postharvest treatment at edible ripeness (CASL and HTCASL) (Figure 2). Hydrothermally treated samples at edible ripeness at both storage times (30 and 50 days, HTCASL30I and HTCASL50I) presented higher contents of CAD, L-AOX, CAAG, and *p*-CAD3 than CASL30I and CASL50I samples (Figure 2). Instead, control—CA samples at edible ripeness (CASL30I and CASL50I) presented higher contents of SAG, *p*-CAD2, TP-UPLC, and *p*-CA than hydrothermally treated samples (STSL30I and STSL50I, Figure 2). A biplot is presented in Figure S2.

**Figure 3:**
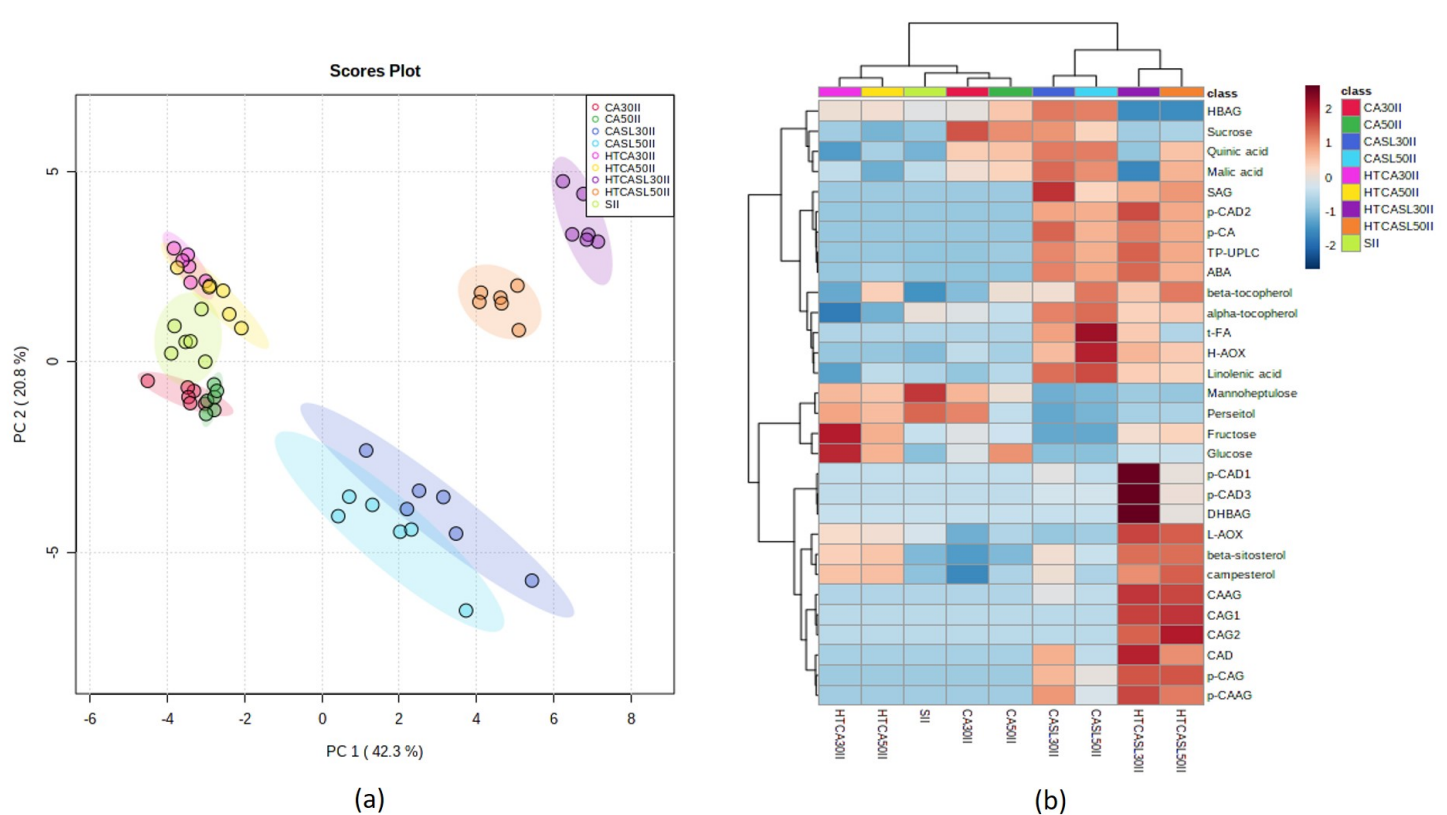
Principal component analysis of the middle harvest dataset. (**a**) Score plot and (**b**) heat map based on hierarchical cluster analysis based on Euclidean distance and Ward algorithm with the 25 significant metabolites revealed by ANOVA followed by the Tukey test (*p* < 0.05). Samples correspond to different treatments, storage times, and corresponding edible ripeness stages. Samples corresponding to the different conditions are on the horizontal axis. SII = Initial day middle harvest; CA30II and CA50II = controlled atmosphere for 30 and 50 days middle harvest; CASL30II and CASL50II = controlled atmosphere for 30 and 50 days middle harvest after shelf-life period; HTCA30II and HTCA50II = hydrothermal treatment for 30 and 50 days middle harvest; HTCASL30II and HTCASL50II = hydrothermal treatment for 30 and 50 days middle harvest after shelf-life period. The average of the 6 replicates is displayed. DM = dry matter, ABA = abscisic acid, H-AOX = hydrophilic antioxidant activity, L-AOX = lipophilic antioxidant activity, DHBAG = dihydroxybenzoic acid glycoside, SAG = syringic acid glycoside, CAG1 = caffeic acid glycoside 1, CAG2 = caffeic acid glycoside 2, HBAG = hydroxybenzoic acid glycoside, *p*-CAG = p-coumaric acid glycoside, CAD = caffeic acid derivative, *p*-CAD1 = *p*-coumaric acid derivative, CAAG = caffeic acid acetilglycoside, *p*-CA = *p*-coumaric acid, *p*-CAAG = *p*-coumaric acid acetylglycoside, *p*-CAD2 = *p*-coumaric acid derivative, *t*-FA = trans-ferulic acid, *p*-CAD3 = *p*-coumaric acid derivative.

For middle harvest fruit (II), the dataset PCA analysis revealed four marked clusters (Figure 3). Samples at edible ripeness, either CA (CASL30II and CASL50II) or hydrothermally treated (HTCASL30II and HTCASL50II), presented higher contents of *p*-CAG, CAD, *p*-CAAG, SAG, *p*-CA, *p*-CAD2, ABA, and TP-UPLC. This dataset, as shown in Figure 3, for the hydrothermally treated (HTCASL30II and HTCASL 50II) samples displayed higher contents of CAAG, CAG1, CAG2, L-AOX, and β-sitosterol than CA treated samples at edible ripeness (CASL30II and CASL50II). A biplot is presented in Figure S3.

## 3. Discussion

### 3.1. Differences at Harvest in Primary and Secondary Metabolites and Antioxidant Activity between Early and Middle Harvest Fruit

Differences in primary and secondary metabolite content and antioxidant activity were observed between early and middle harvests. With the evolution of the harvest, some of these characteristics increased (DM, oil, ABA, and sucrose), others decreased (mannoheptulose, glucose, fructose, quinic acid, and HBAG), while others did not show significant changes (malic acid, fatty acids, tocopherols, phytosterols, and H-AOX and L-AOX). The increase in DM for Peruvian Hass from early (21.39%) to middle (22.38%) harvest has been reported previously [8], with contents very close to those found in this study. In this regard, it should be noted that there are regulations that define a minimum of 21% DM for avocado cv. Hass to be considered to have reached physiological maturity [17]. In addition, in different countries of South America, including Peru, early harvest starts from DM ≥ 19.0% values. The increments in oil content of avocados between harvest stages have been calculated [5,18,19], indicating that oil biosynthesis happens during fruit development [20]. With respect to ABA, this phytohormone has been reported as the main actor in mediating the adaptation of plants to stress and in avocados cv. Fuerte, ABA increased as the harvest stages advanced [21].

A total of six sugars were detected, where mannoheptulose and perseitol (sugars C_7_), together represented ~93% of total sugars. The decrease in both sugars as harvest time or stage advanced has been reported in previous studies [5,8,9,22]. These C_7_ sugars play a central role during avocado growth and development, but confirmation is still needed regarding the biosynthetic routes and the physiological function during avocado growth and development [20]. In addition, mannoheptulose and other C_7_ sugars are involved in the regulation of carbon flux and protection against oxidative damage [23]. Among the organic acids detected, quinic acid decreased as harvest stage advanced. High concentrations of quinic acid have been found at early stages of development in various fruits [24]. The shikimic acid pathway is important in the biosynthesis of a range of secondary plant products, and quinic acid is a side product of this pathway. The molecular and enzymatic control of quinic acid storage and metabolism would be expected to affect metabolites and products of the shikimate pathway, such as aromatic amino acids, folates, and the phenylpropanoid pathway, and it is from this last pathway that phenolic acids can be synthesized: hydroxybenzoic and hydroxycinnamic acids, flavonoids, etc. [24]. Our results suggest that the decrease in quinic acid content is caused by the synthesis of other secondary metabolites, such as phenolic acids. In addition, the decrease in the only phenolic compound detected at harvest, HBAG, might be indicative that other compounds of phenolic nature are being prioritized instead of this compound with the advancement of the harvest stage. 

Lipophilic compounds did not display significant changes as harvest stages advanced. The contents and profile of fatty acids, tocopherols, and phytosterols present in Hass avocados during our study are very close to those reported by other studies [1,2,5,8,25]. As a function of all the results presented, well-documented differences between early and middle harvest fruit were evidenced and explain the different ripening behavior between early and middle harvest fruit in terms of ripening speed, at least when considering the amount of C_7_ sugars, the main respiratory substrates in avocado fruit. 

### 3.2. Changes in Primary and Secondary Metabolites and Antioxidant Activity after Postharvest Treatments and at Edible Ripeness for Early and Middle Harvest Fruit

For both harvest stages (I and II) corresponding to early and middle harvests, among the metabolites that decreased drastically and progressively during avocado storage (CA or HTCA) until edible ripeness were mannoheptulose and perseitol. The decrease found in these sugars indicates that they were being used as primary substrates during respiration. On the contrary, sucrose content in fruit subjected to CA increased until edible ripeness, which was not evident for the HTCA treatment, while in general a higher content of glucose and fructose was found in ready to eat avocados subjected to HTCA than in avocados under CA. Previous studies have reported a downward trend for mannoheptulose and perseitol as they reach edible ripeness, while they did not observe any pattern for the fate of sucrose during ripening [5,8,12,22,26]. The depletion of mannoheptulose from avocado mesocarp during ripening might be an indication of the participation of this sugar as the inhibiting factor of avocado softening [26]. In pomegranate fruits, higher sugar contents of glucose and fructose after heat treatment (dips at 45 °C for 4 min) have also been reported [27]. The presence of sugars in greater quantity in heat-treated avocados could be because the biochemical processes of fruit respiration were temporarily affected, decreasing the respiration rate where sugars as well as organic acids act as the main substrates. 

Defilippi et al. [28] found in Chilean Hass avocados a decrease in the amount of malic, ascorbic, citric, and succinic acids as ripening progressed at 20 °C, with a drastic decrease in malic acid content. Similarly, a decrease in quinic acid during avocado ripening has been reported [21]. In this study, this decreasing trend was not observed since malic and quinic acids did not vary in content compared to the harvest stage. It is possible that the energy required for the realization of the different metabolic processes was more oriented towards the oxidation of carbohydrates (in particular, perseitol and mannoheptulose) than of organic acids.

Previous studies have reported that the harvest stage influences the composition of oil more significantly than postharvest ripening [19], and the fatty acid profile did not change as avocado reached edible ripeness [5,12,14,22]. At edible ripeness, α-tocopherol content in CA treated fruit from both harvests presented higher values than HTCA, which remained close to those at harvest (initial day). These findings indicate that the hydrothermal treatment may have affected the integrity of α-tocopherol. The heat treatment can affect oxidative metabolism, which might explain the reduction in tocopherol concentration [1]. In contrast, at edible ripeness for the two harvests, β-sitosterol increased markedly for HTCA but not for CA. A previous study reported that phytosterol content (composed of β-sitosterol, campesterol, and stigmasterol) increased during ripening [1]. An increase in total phytosterol content associated with fruit senescence suggests that phytosterols participate in the maintenance and function of cell membranes. Different results were reported for Hass avocados after cold storage and at edible ripeness, where phytosterols (β-sitosterol, stigmasterol, and campesterol) did not display any significant change [5].

ABA is an important compound in the avocado ripening process. This hormone accumulates in the mesocarp during ripening, alongside an increase in ethylene biosynthesis [29]. The same behavior was evidenced in this study, with ABA displaying the highest contents at edible ripeness with no significant differences between CA and HTCA fruit. In Fuerte avocado, an increase in ABA content has been shown to reach its maximum concentration at edible ripeness [30]. Avocado mature fruit with lower water content ripened faster, possibly because the fruit was more stressed and contained a higher endogenous ABA concentration [11]. The closely aligned amounts of ABA found at edible ripeness for both CASL and HTCASL samples might be due to similar DM values. 

The identification of phenolic compounds was based on chromatographic and spectral data such as retention time, *m*/*z* ratio, λ*max*, [MH] and fragments (% abundance) previously evaluated for Peruvian Hass avocados cold stored in regular air conditions and ripened at shelf-life conditions [5], where a total of 19 phenolic compounds were detected and identified, of which 14 coincided with those found in this study. The same authors reported that at edible ripeness the number and quantity of phenolic compounds considerably increased, especially in *p*-coumaric and caffeic acids and their derivatives; a similar trend in the evolution of phenolic compounds with the transition from green to ripe stages for six different avocado cultivars including Hass has previously been reported [31,32]. Among the detected phenolic compounds in Hass avocado, epicatechin, *p*-coumaric, and ferulic acid, among others, were found [31], while epicatechin was not detected in this study. Other striking aspects extracted from the profile and quantity of phenolic compounds detected in avocados (Table 3) at edible ripeness were (1) only CA stored fruit presented a lower number of phenolics (11) with respect to HTCA (14), (2) TP-UPLC was higher for CA than HTCA fruit of early harvest avocados with no differences between CA and middle harvest HTCA, (3) middle harvest avocados presented higher amounts (mg/kg DM) of phenolic compounds than early harvest and (4) the amount of phenolics found in descending order for CASL was as follows: *p*-coumaric acids > hydroxybenzoic acids > syringic acid > caffeic acids ~ ferulic acid; and for HTCASL was as follows: *p*-coumaric acids > caffeic acids > hydroxybenzoic acids > syringic acid > ferulic acid. The differences found could be based on the modulation of the metabolism of phenolic compounds in response to the conditions to which the fruit were exposed. Thus, the hydrothermal treatment may have influenced some of the metabolites present, with this being more pronounced for middle harvest fruit. In agreement with the results of our study, it was observed that thermal stress induces the accumulation of phenolics in plants by activating their biosynthesis as well as inhibiting their oxidation [32,33].

Finally, increased H-AOX at edible ripeness has been reported in Hass avocado [5], but the existing literature also reports an invariable H-AOX during ripening evaluated through different antioxidant reaction mechanisms (DPPH, TEAC, and ORAC), while the lipophilic antioxidant activity varied depending on the reaction mechanism (increased with DPPH, decreased with TEAC, and remained constant with ORAC) [4]. The hydrothermal treatment may have caused the development of oxidative stress, triggering both hydrophilic and lipophilic antioxidant systems, which were not evaluated in this study, such as antioxidant enzymes or other antioxidant molecules, which had an important effect on the antioxidant properties detected. Heat treated pomegranate fruits (dips at 45 °C for 4 min) and stored at 2 °C for 90 days exhibited higher total antioxidant activity than control fruit and was primarily correlated with the high levels of total phenolics [27].

## 4. Materials and Methods

### 4.1. Fruit Material 

Avocados cv. Hass corresponding to early (~20% DM, mid-April 2018) and middle (~23 DM, end-May 2018) harvest stages (I and II) were collected from ten trees from Fundo Jorge Bustamante, Imperial district, Cañete province, Peru (40 m.a.s.l, south latitude: 13°03′40′′ and west longitude: 76°21′11′′) and immediately transported to the laboratory. Fruit (220–320 g) were cleaned and allowed to stand overnight at ~20 ± 1 °C until postharvest treatments and conditions were applied. 

### 4.2. Postharvest Treatments and Storage Conditions

Fruit from both harvest stages (early and middle), were subjected to a hydrothermal treatment (38 °C for 1 h) or not (control) and stored in controlled atmosphere conditions corresponding to 4 kPa O_2_ and 6 kPa CO_2_ at 7 °C and ~80% RH for 30 and 50 days. After 30 and 50 days, both control fruit (CA) and hydrothermally treated (HTCA) avocados were subjected to shelf conditions (20 ± 1 °C and 70 ± 5% RH) until edible ripeness was attained (firmness 4–8 N) [12]. Fruit were sampled at harvest, after 30 and 50 days of storage conditions, and at their corresponding storage times at edible ripeness. Edible ripeness for the early harvest for the CA30, CA50, HTCA30, and HTCA50 samples was reached at ~17, 11, 14, and 12 days, respectively, while for the middle harvest the values were ~14, 11, 13, and 10 days, respectively. Avocados did not show any physiological internal or external damage at edible ripeness. Per sampling point, six independent fruit (six biological replicates) were taken, the mesocarp was pulverized in liquid nitrogen, ground, packaged in polyethylene bags, and stored at −80 °C until analysis [5].

### 4.3. Determination of Relevant Primary and Secondary Metabolites

#### 4.3.1. Determination of Dry Matter and Oil Content

Dry matter (DM) was determined according to the AOAC 920.151 method [34]. Oil content was determined by the Soxhlet method, AOAC method 2003.05 [34]. Results were expressed as percentages (%).

#### 4.3.2. Determination of Sugars and Organic Acids

Sugars and organic acids were extracted following the methodology described by Hatoum et al. [35] with slight modifications. Briefly, 100 mg of avocado pulp was mixed with 1000 μL of cold methanol (0 °C), 750 μL of chloroform, and 1500 μL of Milli-Q ultrapure water. The mixture was stirred at 70 °C for 15 min and then centrifuged at 6600× *g* at 4 °C for 15 min (Eppendorf 5430R, Hamburg, Germany). The polar phase was recovered and diluted with acetonitrile, then samples were passed through a PVDF 0.22 μm Millipore filter, and stored at −80 °C until analysis. Chromatographic analysis was performed on a UHPLC UltiMate 3000 chromatograph (Thermo Scientific, MO, USA)). The separation was carried out on a Shodex HILICpak VG-50 2D column (150 mm × 2 mm, 5 µm) at a flow of 0.25 mL min^−1^ and 40 °C. The mobile phase was composed of 0.5% aqueous ammonia (A) and acetonitrile (B). The following gradient was applied: 80% B from 0 to 2 min, then increased to 10% B at 10 min and maintained for 3 min, then for 0.5 min at 80% B and for 10 min at 80% B. The injection volume was 5 µL. Detection was performed on a TSQ Quantum Access Max triple quadrupole mass spectrometer (Thermo Scientific, CA, USA) equipped with an ionization source (H-ESI). Source parameters corresponded to: −4 kV ionization voltage, 200 °C vaporizer temperature, 300 °C capillary tube temperature. SIM mode was used. Each compound was separated according to the molecular ion ([M-H]^-^). The data were acquired using the Chromeleon ™ and TSQ Tune ™ software and later processed with the Xcalibur™ program (ThermoScientific, CA, USA). The identification and quantification were carried out based on previously injected standards for glucose, fructose, sucrose, mannoheptulose, perseitol, malic acid, and quinic acid. Results were expressed as g/kg of dry matter (DM).

#### 4.3.3. Fatty Acid Content and Profile

The fatty acids of avocado oil samples were converted into methyl esters according to the method proposed by Meurens et al. [36] with slight modifications. The fatty acid methyl esters were separated, identified, and quantified using a gas chromatograph (GC Plus, Shimadzu, GC-2010 model, Tokyo, Japan) equipped with a flame ionization detector (FID) following the methodology proposed by Campos et al. [5]. The identification and quantification were carried out based on previously injected standards for oleic, palmitic, palmitoleic, linoleic, and α-linolenic acids. The concentration of each fatty acid was expressed as the percentage (%) of total fatty acids in the avocado oil.

#### 4.3.4. Tocopherol Content and Profile

The samples were prepared following the methodology reported by Amaral et al. [37] with slight modifications. The analysis was performed by high performance liquid chromatography (Waters 2695 Separation Module, Waters, Milford, MA, USA) with fluorescence detection as reported by Chirinos et al. [38]. The results were expressed in mg/kg DM.

#### 4.3.5. Phytosterol Content and Profile

Preparation of samples were realized as reported by Chirinos et al. [38]. Phytosterol composition was determined by gas chromatography (GC Plus, Shimadzu, GC-2010 model, Tokyo, Japan) coupled with FID according to Campos et al. [5]. Results were expressed as g/kg DM.

#### 4.3.6. Determination of Hydrophilic and Lipophilic Antioxidant Activity

The extracts to determine H-AOX and L-AOX were obtained as follows. Seven grams of mesocarp was mixed with 20 mL of dichloromethane under stirring for 30 min at room temperature. The mixture was subjected to centrifugation at 2500× *g* for 15 min, then the supernatant (lipophilic extract) was recovered and stored at −80 °C until use. The residual cake was subjected to extraction with 10 mL of 80% (*v*/*v*) methanol under stirring for 60 min at room temperature, then the mixture was centrifuged at 2500× *g* for 15 min, and the supernatant (hydrophilic extract) was recovered. The extraction was repeated with 10 mL of the same solvent and the extracts were mixed and stored at −80 °C until use. The ABTS assay, for the evaluation of both antioxidant activities, was performed as previously described by Arnao et al. [39] and Campos et al. [5]. The antioxidant activity was calculated as µmol of Trolox equivalents (TE)/g DM from a standard curve developed with Trolox.

#### 4.3.7. Abscisic Acid Content by UPLC-PDA

The extract used to analyze abscisic acid (ABA) was the same as that used for the analysis of H-AOX. ABA was quantified using the UPLC Acquity H-Class (Waters) system coupled to a PDA detector (eλ detector) and Empower II software. An Acquity BEH C18 column (1.7 µm, 100 mm × 2.1 mm) (Waters) with a BEH C18 column guard (1.7 µm, 5 mm × 2.1 mm) was used. The mobile phase was composed of (A) 0.1% formic acid in MilliQ water and (B) acetonitrile with 0.1% formic acid. The gradient used was as follows: 2% B for 2 min, 2–7% B in 2 min, 7–12% B in 11 min, 12–26% B in 5 min, 26–55% B in 5 min, and 95% B for 3 min and the column was equilibrated with 2% B for 5 min. The injected volume was 10 μL with a flow of 0.2 mL/min and a column temperature of 30 °C. ABA was identified and quantified by comparing their retention time and UV-visible spectral data with a previously known injected standard (260 nm). The results were expressed in mg/kg DM.

#### 4.3.8. UPLC-MS-MS/PDA/-qToF Analysis of Phenolic Compounds Content and Profile

Prior to analysis, phenolic compounds were extracted using the same procedure as described above for H-AOX, but the mesocarp was previously defatted with hexane. Hydrophilic extracts were then concentrated at vacuum (~40 °C) until dryness and resuspended with methanol MS grade. Before analysis, methanolic extracts were filtered through a PVDF 0.22 μm Millipore and stored at −80 °C until analysis. Phenolic compounds were detected and identified as reported by Campos et al. [5] using an UPLC (ACQUITY UPLC I-Class, Waters Corp., Milford, MA, USA) equipped with an Acquity BEH column (1.7 μm, 100 mm × 2.1 mm, Waters Corp) connected to a quadrupole time-of-flight mass spectrometer (Xevo G2-XS QTof, Waters Corp). The mobile phase was composed of 5% formic acid (A) and acetonitrile with 0.1% formic acid (B), and the solvent gradient was as follows: 2% B for 0.5 min, then in 10 min to 15% B, then in 1 min to 98% B and kept for 1.5 min, then in 0.5 min to 98% and re-equilibrated for 1.5 min. The injection volume was 1 μL, the flow rate was set to 0.3 mL min^−1^, and the column temperature at 30 °C. Mass spectra were acquired continuously using electrospray ionization in positive mode (3 kV) in MS/MS mode with a scan time of 0.5 s. The desolvation temperature was 300 °C, the cone gas flow was 20 L h^−1^, and the desolvation gas flow was 1000 L/h. The MS/MS fragmentation process was accomplished at normalized collision energy ramped from 6 to 20 eV. The accurate mass and elemental composition of the precursor and fragment ions were calculated and sequenced using MassLynx software (Waters Corp., Milford, MA, USA). Monoisotopic mass of the molecular ion [M+] with a mass error < 5 ppm and fragmentation patterns were used for identification by comparison with literature references and online databases (PubChem) and with known UV-Vis data of previously injected standards: caffeic, *p*-coumaric, sinapic, trans-ferulic hydroxybenzoic, and syringic acids (Sigma Chemical Co., St Louis, MO, USA). Quantification was performed based on the main aglycon of the derived phenolic compound with previously developed standard curves. The results were expressed in mg/kg DM.

### 4.4. Statistical Analysis

The results were reported as mean ± standard deviation of six independent biological replicates (six independent fruit). Analysis of variance (ANOVA) was used to compare the means, and Tukey’s test was used to assess statistically significant differences among treatments (*p* < 0.05). All statistical analyses were performed with Statgraphics Centurion XV (Stat Point Technologies, Inc., Warrenton, VA, USA). Principal component analysis (PCA) and hierarchical clustering analysis (using Euclidean distance and the Ward algorithm) were performed on the normalized data using MetaboAnalyst 4.0 (Xia Lab, McGill University, Montréal, QC, Canada).

## 5. Conclusions

A hydrothermal treatment prior to CA storage (HTCA) for long distance markets (up to 50 days) did not negatively impact the level of primary and secondary metabolites. Different behaviors were observed among the studied metabolites from harvest to edible ripeness. In general, mannoheptulose and perseitol decreased while β-sitosterol, ABA, H-AOX, L-AOX, and total phenolic compounds evaluated by UPLC-MS/MS significantly increased (*p* < 0.05). Meanwhile, malic acid, β-tocopherol, campesterol, and fatty acid profiles remained constant. At edible ripeness, the hydrothermally treated fruit presented higher contents of mannoheptulose, perseitol, glucose, fructose, β-sitosterol, and L-AOX, caffeic acid glycoside, caffeic acid derivatives, caffeic acid acetylglycoside, and *p*-coumaric acid derivatives, while the CA-control samples presented higher contents of sucrose, α-tocopherol, H-AOX, hydroxybenzoic acid glucoside, and syringic acid glycoside. Other studied metabolites presented similar values for both CA and HTCA. The length of the postharvest storage (30 or 50 days) for CA and HTCA fruit mostly did not affect their final concentrations. In addition, benzoic acids mainly represented by hydroxybenzoic acid glycoside were the most representative phenolic compound at harvest and after CA or HTCA storage, while cinnamic acids, on the contrary, were synthesized and participated in greater numbers and quantities at edible ripeness, highlighting *p*-coumaric acid derivatives. Interestingly, at edible ripeness there was a higher number of phenolics in the HTCA than the CA treatment, but this was dependent on the harvest stage (higher contents for middle harvest fruit), which might be of relevance in terms of functional compounds the consumer ingests. The results found in this study offer important information for the Hass avocado industry regarding the evolution of the main metabolites in Peruvian Hass avocados subjected to CA and HTCA long storage (up to 50 days) simulating exports to distant markets. 

## Figures and Tables

**Table 1:** Content of dry matter, oil, sugars, and organic acids in Hass avocado from two harvests (early and middle) at harvest (initial), after controlled atmosphere storage, and with previous hydrothermal treatment followed by controlled atmosphere storage, and at their corresponding edible ripeness.

						Hydrothermal Treatment Followed by Controlled Atmosphere			
Compound/ Harvest	Initial (0 day)	Storage (day) CA		Shelf-Life—Edible Ripeness CASL		Storage (day) HTCA		Shelf-Life—Edible Ripeness HTCASL	
		30	50	30 *	50 *	30	50	30 *	50 *
Dry matter (g/100 g DM)									
Early (I)	20.08 ± 0.77 ^aB^	22.15 ± 1.22 ^aB^	22.23 ± 1.00 ^aB^	22.42 ± 3.14 ^aA^	21.01 ± 2.09 ^aA^	20.26 ± 1.68 ^aB^	21.51 ± 1.64 ^aA^	21.58 ± 1.45 ^aB^	20.50 ± 1.37 ^aA^
Middle (II)	22.95 ± 1.00 ^aA^	24.13 ± 1.21 ^aA^	23.62 ± 0.64 ^aA^	22.88 ± 1.59 ^aA^	22.93 ± 1.79 ^aA^	24.35 ± 1.37 ^aA^	24.94 ± 2.11 ^aA^	22.92 ± 1.08 ^aAB^	21.98 ± 0.79 ^aA^
Oil (g/100 g DM)									
Early (I)	10.47 ± 0.97 ^aB^	11.99 ± 0.57 ^aB^	12.72 ± 1.29 ^aB^	13.20 ± 2.03 ^aA^	12.24 ± 2.21 ^aA^	10.59 ± 1.64 ^aB^	11.18 ± 1.29 ^aB^	12.96 ± 1.22 ^aB^	11.26 ± 1.99 ^aA^
Middle (II)	13.31 ± 1.49 ^aA^	14.47 ± 0.96 ^aA^	14.57 ± 0.73 ^aA^	14.87 ± 1.58 ^aA^	14.45 ± 1.44 ^aAA^	15.33 ± 0.37 ^aA^	15.84 ± 1.06 ^aA^	14.66 ± 1.05 ^aAB^	14.57 ± 1.05 ^aAB^
Sugars (g/kg DM)									
Mannoheptulose									
Early (I)	87.40 ± 15.89 ^aA^	66.61 ± 18.72 ^abA^	37.84 ± 1.16 ^cA^	1.09 ± 0.37 ^eA^	7.28 ± 6.38 ^dA^	56.31 ± 14.14 ^bA^	48.31 ± 18.04 ^bA^	0.52 ± 0.18 ^eB^	4.13 ± 1.73 ^dAB^
Middle (II)	54.08 ± 18.70 ^aB^	34.47 ± 10.02 ^abB^	23.50 ± 2.08 ^abA^	1.52 ± 0.13 ^dA^	3.24 ± 1.74 ^dA^	33.72 ± 5.45 ^abB^	31.18 ± 13.40 ^abAB^	8.05 ± 1.19 ^cA^	6.58 ± 0.72 ^cA^
Perseitol									
Early (I)	32.62 ± 7.06 ^aA^	26.99 ± 8.28 ^abA^	11.57 ± 1.73 ^cA^	0.41 ± 0.01 ^eA^	6.22 ± 2.73 ^dA^	17.52 ± 5.46 ^bcA^	20.41 ± 2.98 ^bA^	0.25 ± 0.10 ^efB^	10.47 ± 1.90 ^cA^
Middle (II)	22.03 ± 6.57 ^aAB^	20.20 ± 6.73 ^aA^	9.06 ± 0.71 ^cA^	3.46 ± 2.44 ^dB^	4.45 ± 0.21 ^dA^	18.15 ± 1.42 ^aA^	16.40 ± 3.54 ^abAB^	7.30 ± 0.88 ^cA^	7.60 ± 2.04 ^cAB^
Sucrose									
Early (I)	2.27 ± 0.76 ^cAB^	5.12 ± 1.13 ^abB^	6.13 ± 1.23 ^aB^	1.84 ± 0.68 ^cB^	7.03 ± 3.00 ^aA^	ND	3.06 ± 1.00 ^b^^cA^	ND	3.26 ± 0.83 ^b^^cA^
Middle (II)	3.59 ± 0.42 ^dA^	11.58 ± 0.65 ^aA^	9.93 ± 0.51 ^bA^	9.68 ± 5.46 ^abA^	7.57 ± 1.37 ^bcA^	3.89 ± 0.23 ^d^	2.81 ± 0.66 ^deA^	3.92 ± 0.05 ^d^	4.21 ± 0.13 ^dA^
Glucose									
Early (I)	2.34 ± 0.58 ^b^	1.30 ± 0.43 ^cdA^	4.93 ± 0.94 ^aA^	2.15 ± 0.59 ^bc^	0.55 ± 0.34 ^d^	4.23 ± 0.91 ^aA^	4.28 ± 0.53 ^aA^	0.73 ± 0.13 ^dA^	2.03 ± 0.22 ^bA^
Middle (II)	ND	1.06 ± 1.04 ^abA^	2.58 ± 0.81 ^abB^	ND	ND	3.74 ± 0.69 ^aA^	2.18 ± 0.75 ^abB^	0.78 ± 0.18 ^b^^cA^	0.80 ± 0.08 ^cB^
Fructose									
Early (I)	3.37 ± 0.83 ^bA^	1.84 ± 0.44 ^cA^	3.98 ± 0.16 ^bA^	1.38 ± 0.16 ^cd^	0.87 ± 0.20 ^d^	5.05 ± 0.76 ^aA^	4.65 ± 0.41 ^abA^	1.88 ± 0.33 ^cA^	2.99 ± 0.29 ^bA^
Middle (II)	1.06 ± 0.46 ^cB^	1.37 ± 0.40 ^cA^	1.15 ± 0.09 ^cB^	ND	ND	3.75 ± 0.75 ^aB^	2.35 ± 0.06 ^bB^	1.67 ± 0.26 ^cAB^	1.90 ± 0.23 ^cB^
Organic acids (g/kg DM)									
Malic acid									
Early (I)	7.43 ± 0.97 ^aA^	8.70 ± 1.20 ^aA^	7.90 ± 0.72 ^aA^	7.90 ± 1.50 ^aA^	8.98 ± 0.94 ^aA^	4.69 ± 0.83 ^bA^	5.64 ± 0.35 ^abA^	5.13 ± 1.43 ^abA^	7.56 ± 1.24 ^aA^
Middle (II)	6.88 ± 0.02 ^bA^	7.62 ± 0.29 ^abA^	7.92 ± 0.17 ^abA^	9.19 ± 0.61 ^aA^	8.79 ± 0.19 ^aA^	6.90 ± 0.17 ^abA^	6.03 ± 1.11 ^abA^	5.45 ± 0.06 ^bA^	8.35 ± 1.77 ^aA^
Quinic acid									
Early (I)	2.14 ± 0.61 ^abA^	2.05 ± 0.61 ^abA^	1.78 ± 0.35 ^abA^	2.68 ± 0.86 ^aA^	2.27 ± 0.71 ^abA^	1.93 ± 0.73 ^aA^	1.83 ± 0.35 ^aA^	2.10 ± 0.40 ^aA^	2.41 ± 0.24 ^aA^
Middle (II)	0.68 ± 0.02 ^bB^	1.24 ± 0.08 ^aA^	1.29 ± 0.04 ^aA^	1.54 ± 0.14 ^aA^	1.53 ± 0.22 ^aA^	0.58 ± 0.08 ^bB^	0.84 ± 0.13 ^bB^	0.77 ± 0.05 ^bB^	1.29 ± 0.36 ^abB^

The values in each row correspond to the mean value of six independent determinations (six avocados) ± standard deviation (n = 6). Different lowercase superscript letters in the same row indicate significant differences. Letters in different uppercase superscripts in the same column per compound indicate significant difference (*p* < 0.05) determined by a Tukey test. ND = Not detected. (*) correspond to ready to eat fruit (edible ripeness) subjected to ripening at shelf-life conditions (20 °C) after 30 and 50 days of controlled atmosphere or hydrothermal treatment followed by controlled atmosphere storage.

**Table 2:** Content of fatty acids, tocopherols, phytosterols, abscisic acid, and hydrophilic and lipophilic antioxidant activity in Hass avocado from two harvests (early and middle) at harvest (initial), after controlled atmosphere storage, and with previous hydrothermal pretreatment followed by controlled atmosphere storage, and at their corresponding edible ripeness.

		Controlled Atmosphere				Hydrothermal Treatment Followed by Controlled Atmosphere			
Compound/ Harvest	Initial (0 day)	Storage (day) CA		Shelf-Life—Edible Ripeness CASL		Storage (day) HTCA		Shelf-Life—Edible Ripeness HTCASL	
		30	50	30 *	50 *	30	50	30 *	50 *
Fatty acids (%)									
Palmitic									
Early (I)	30.09 ± 1.05 ^aA^	29.51 ± 1.91 ^aA^	29.30 ± 1.16 ^aA^	28.32 ± 0.81 ^abA^	26.50 ± 2.22 ^bA^	29.77 ± 1.85 ^abA^	30.76 ± 1.67 ^aA^	27.88 ± 1.33 ^bA^	28.26 ± 1.53 ^abA^
Middle (I)	29.35 ± 0.83 ^aA^	29.15 ± 2.22 ^aA^	28.66 ± 0.69 ^aA^	27.36 ± 2.26 ^aA^	26.93 ± 3.25 ^aA^	27.52 ± 0.88 ^abAB^	26.79 ± 1.26 ^bB^	27.40 ± 1.89 ^abA^	26.96 ± 1.84 ^abAB^
Palmitoleic									
Early (I)	14.38 ± 0.65 ^aA^	15.23 ± 1.09 ^aA^	15.80 ± 0.72 ^aA^	14.07 ± 1.06 ^aA^	14.74 ± 2.13 ^aA^	14.41 ± 1.99 ^aA^	14.84 ± 0.62 ^aA^	14.43 ± 1.62 ^aA^	14.57 ± 1.76 ^aAB^
Middle (II)	15.34 ± 1.09 ^aA^	15.24 ± 1.19 ^aA^	15.94 ± 1.05 ^aA^	14.53 ± 0.96 ^aA^	15.09 ± 2.41 ^aA^	15.23 ± 1.48 ^aA^	15.02 ± 2.09 ^aA^	15.34 ± 1.19 ^aA^	15.75 ± 1.06 ^aA^
Oleic									
Early (I)	40.01 ± 1.17 ^aA^	39.55 ± 1.85 ^aA^	37.87 ± 1.83 ^aA^	41.37 ± 1.42 ^aA^	41.98 ± 4.65 ^aA^	41.66 ± 3.75 ^aA^	39.22 ± 1.03 ^aAB^	41.86 ± 2.53 ^aA^	40.26 ± 2.02 ^aB^
Middle (II)	40.25 ± 2.26 ^aA^	41.40 ± 4.47 ^aA^	39.80 ± 1.84 ^aA^	42.76 ± 2.53 ^aA^	41.20 ± 5.19 ^aA^	42.66 ± 1.89 ^aA^	43.31 ± 2.53 ^aA^	41.84 ± 2.22 ^aA^	42.22 ± 2.68 ^aB^
Linoleic									
Early (I)	14.83 ± 1.37 ^aA^	14.99 ± 1.50 ^aA^	16.28 ± 1.09 ^aA^	15.36 ± 1.05 ^aA^	15.87 ± 1.83 ^aA^	14.80 ± 1.79 ^aA^	14.51 ± 1.22 ^aA^	15.02 ± 1.86 ^aA^	15.99 ± 1.61 ^aA^
Middle (II)	14.38 ± 1.18 ^abA^	13.59 ± 1.63 ^abA^	14.96 ± 0.82 ^abAB^	14.58 ± 1.65 ^abA^	15.99 ± 1.27 ^aA^	14.02 ± 0.79 ^aAB^	14.26 ± 0.99 ^aA^	14.73 ± 0.81 ^aA^	14.40 ± 1.49 ^aAB^
α-linolenic									
Early (I)	0.77 ± 0.06 ^aA^	0.70 ± 0.09 ^aA^	0.72 ± 0.07 ^aA^	0.86 ± 0.19 ^aA^	0.88 ± 0.18 ^aA^	0.73 ± 0.17 ^abA^	0.67 ± 0.07 ^bA^	0.82 ± 0.14 ^abA^	0.93 ± 0.18 ^aA^
Middle (II)	0.67 ± 0.08 ^aA^	0.60 ± 0.05 ^aAB^	0.62 ± 0.04 ^aA^	0.75 ± 0.10 ^aA^	0.77 ± 0.13 ^aA^	0.57 ± 0.05 ^aAB^	0.63 ± 0.06 ^aA^	0.69 ± 0.07 ^aAB^	0.69 ± 0.07 ^aB^
Tocopherols (mg/kg DM)									
α-tocopherol									
Early (I)	61.06 ± 10.79 ^bcB^	61.78 ± 6.80 ^bcB^	64.89 ± 8.46 ^bcB^	81.37 ± 4.68 ^aA^	83.45 ± 11.92 ^aA^	54.03 ± 7.15 ^cB^	53.22 ± 5.52 ^cB^	78.13 ± 8.90 ^aA^	52.57 ± 3.00 ^cB^
Middle (II)	72.56 ± 5.74 ^bAB^	70.71 ± 24.87 ^abAB^	67.59 ± 11.70 ^abA^	85.39 ± 6.14 ^aA^	87.43 ± 9.07 ^aA^	52.35 ± 3.66 ^cB^	59.59 ± 5.67 ^cAB^	76.40 ± 9.32 ^bA^	77.36 ± 5.18 ^bA^
β-tocopherol									
Early (I)	20.74 ± 2.35 ^bcA^	19.15 ± 3.80 ^cB^	29.01 ± 4.96 ^abA^	23.35 ± 2.70 ^ab cAB^	27.56 ± 7.68 ^abA^	16.31 ± 2.46 ^cB^	20.83 ± 1.52 ^bcB^	25.54 ± 3.42 ^abA^	21.50 ± 3.19 ^bcB^
Middle (II)	21.57 ± 4.05 ^bA^	23.54 ± 10.16 ^abAB^	27.69 ± 3.89 ^abA^	27.85 ± 4.39 ^abA^	32.23 ± 6.70 ^aA^	22.58 ± 3.64 ^bAB^	28.93 ± 3.54 ^abA^	29.32 ± 2.61 ^abA^	32.18 ± 1.90 ^aA^
Phytosterols (g/kg DM)									
β-sitosterol									
Early (I)	155.67 ± 15.05 ^abA^	141.87 ± 10.55 ^bA^	163.38 ± 10.31 ^abA^	166.38 ± 12.93 ^abA^	177.72 ± 32.90 ^aA^	148.30 ± 14.35 ^abB^	147.06 ± 10.93 ^abB^	190.44 ± 20.11 ^aB^	179.57 ± 10.97 ^aAB^
Middle (II)	150.08 ± 11.06 ^bA^	142.55 ± 15.02 ^bA^	149.68 ± 10.91 ^abAB^	173.52 ± 15.12 ^aA^	163.10 ± 17.55 ^abA^	179.05 ± 4.83 ^aA^	182.01 ± 10. 68 ^aA^	198.10 ± 3.80 ^aA^	198.54 ± 9.15 ^aA^
Campesterol									
Early (I)	24.78 ± 1.71 ^aA^	24.41 ± 1.49 ^aA^	26.19 ± 1.86 ^aAB^	27.95 ± 2.80 ^aA^	26.71 ± 5.02 ^aA^	24.82 ± 0.93 ^bB^	23.90 ± 1.52 ^bB^	28.98 ± 1.63 ^aA^	26.06 ± 1.76 ^bAB^
Middle (II)	25.43 ± 1.90 ^aA^	24.06 ± 1.72 ^aA^	25.99 ± 2.02 ^abAB^	27.40 ± 1.47 ^aA^	25.99 ± 2.02 ^aA^	28.31 ± 1.03 ^aA^	28.39 ± 0.92 ^aA^	29.33 ± 1.70 ^aA^	30.13 ± 0.97 ^aA^
Abscisic acid (mg/kg DM)									
Early (I)	1.79 ± 0.40 ^cA^	6.01 ± 0.97 ^bA^	7.13 ± 0.23 ^bA^	22.36 ± 3.84 ^aA^	19.64 ± 4.03 ^aA^	2.52 ± 0.10 ^bA^	3.17 ± 1.66 ^bA^	24.41 ± 3.21 ^aA^	25.49 ± 0.05 ^aA^
Middle (II)	2.37 ± 0.30 ^cAB^	2.16 ± 0.06 ^cB^	2.13 ± 0.19 ^cB^	24.36 ± 9.86 ^aA^	20.72 ± 5.25 ^aA^	2.47 ± 1.39 ^bcA^	3.92 ± 1.02 ^bA^	26.76 ± 1.56 ^aA^	19.73 ± 1.03 ^aB^
H-Antioxidant activity (µmol/g TE DM)									
Early (I)	16.96 ± 2.12 ^bA^	18.74 ± 1.79 ^abA^	18.18 ± 1.60 ^abA^	19.50 ± 2.27 ^abA^	21.25 ± 3.44 ^aA^	15.47 ± 2.30 ^bA^	16.47 ± 2.25 ^bA^	21.75 ± 2.95 ^aA^	20.47 ± 2.63 ^abA^
Middle (II)	14.60 ± 1.97 ^bA^	16.60 ± 2.67 ^bA^	15.77 ± 1.04 ^bB^	20.50 ± 4.23 ^abA^	25.37 ± 3.22 ^aA^	15.13 ± 1.74 ^bA^	15.10 ± 1.3 ^bA^	20.74 ± 2.88 ^aA^	19.85 ± 2.44 ^aA^
L-Antioxidant activity (µmol/g TE DM)									
Early (I)	2.44 ± 0.14 ^bA^	2.59 ± 0.15 ^bA^	2.46 ± 0.40 ^bA^	3.10 ± 0.44 ^abA^	3.45 ± 0.56 ^aA^	3.98 ± 0.60 ^abA^	3.77 ± 0.58 ^abA^	4.28 ± 0.45 ^abA^	4.63 ± 0.58 ^aA^
Middle (II)	2.85 ± 0.51 ^bA^	2.18 ± 0.18 ^bA^	2.58 ± 0.25 ^bA^	2.39 ± 0.30 ^bB^	2.47 ± 0.51 ^bB^	3.18 ± 0.10 ^abB^	3.11 ± 0.31 ^abB^	4.27 ± 0.36 ^aA^	4.11 ± 0.67 ^aAB^

The values in each row correspond to the mean of six independent determinations (six avocados) ± standard deviation (n = 6). Different lowercase superscript letters in the same row indicate significant differences. Letters in different uppercase superscripts in the same column per compound indicate significant difference (*p* < 0.05) by the Tukey test. (*) correspond to ready to eat fruit (edible ripeness) subjected to ripening at shelf-life conditions (20 °C) after 30 and 50 days of controlled atmosphere storage or hydrothermal treatment followed by controlled atmosphere storage.

**Table 3:** Main phenolic compounds content determined by LC-MS ^e^ and PDA in Hass avocado from two harvests (early and middle) at harvest (initial), after controlled atmosphere storage, and with previous hydrothermal pretreatment followed by controlled atmosphere storage, and at their corresponding edible ripeness.

			Controlled Atmosphere				Hydrothermal Treatment Followed by Controlled Atmosphere			
Peak N°	Phenolic Compound (mg/kg DM)/ Harvest Stage	Initial (0 day)	Storage (day) CA		Shelf-Life—Edible Ripeness CASL		Storage (day) HTCA		Shelf-Life—Edible Ripeness HTCASL	
			30	50	30 *	50 *	30	50	30 *	50 *
1	Dihydroxybenzoic acid glycoside (DHBAG)									
	Early (I)	0.0	0.0	0.0	0.0	0.0	0.0	0.0	Tr	Tr
	Middle (II)	0.0	0.0	0.0	0.0	0.0	0.0	0.0	7.28 ± 1.37 ^a^	1.12 ± 0.09 ^b^
2	Syringic acid glycoside (SAG)									
	Early (I)	Tr	Tr	Tr	10.53 ± 5.40 ^aA^	16.20 ± 0.61 ^aA^	Tr	Tr	7.76 ± 0.33 ^bB^	4.33 ± 0.92 ^cB^
	Middle (II)	0.0	0.0	0.0	35.68 ± 3.95 ^aB^	16.82 ± 3.45 ^cA^	Tr	Tr	22.20 ± 0.61 ^bA^	25.04 ± 1.09 ^bA^
3	Caffeic acid glycoside 1 (CAG1)									
	Early (I)	0.0	0.0	0.0	Tr	Tr	0.0	0.0	0.34 ± 0.11 ^bB^	2.47 ± 0.52 ^aB^
	Middle (II)	0.0	0.0	0.0	0.0	0.0	0.0	0.0	21.57 ± 1.88 ^aA^	22.54 ± 1.37 ^aA^
4	Caffeic acid glycoside 2 (CAG2)									
	Early (I)	0.0	0.0	0.0	Tr	Tr	0.0	0.0	Tr	Tr
	Middle (II)	0.0	0.0	0.0	0.0	0.0	0.0	0.0	6.97 ± 1.23 ^a^	8.77 ± 0.29 ^a^
5	Hydroxybenzoic acid glycoside (HBAG)									
	Early (I)	102.73 ± 1.86 ^bA^	85.77 ± 13.34 ^cA^	71.23 ± 2.09 ^cdA^	100.63 ± 3.44 ^bA^	43.49 ± 1.63 ^eB^	107.88 ± 0.71 ^aA^	82.45 ± 5.67 ^cA^	54.77 ± 3.41 ^e^	59.65 ± 3.66 ^e^
	Middle (II)	56.18 ± 14.13 ^cB^	68.66 ± 11.96 ^bcA^	88.62 ± 1.08 ^bA^	117.64 ± 18.57 ^aA^	115.02 ± 5.60 ^aA^	73.01 ± 1.18 ^cB^	74.54 ± 11.81 ^cA^	Tr	Tr
6	*p*-Coumaric acid glycoside (*p*-CAG)									
	Early (I)	0.0	Tr	Tr	12.19 ± 0.71 ^bB^	41.44 ± 0.42 ^aA^	0.0	0.0	43.11 ± 1.84 ^aB^	7.22 ± 1.77 ^cB^
	Middle (II)	0.0	0.0	0.0	55.79 ± 5.84 ^bA^	31.57 ± 1.73 ^cA^	0.0	0.0	81.98 ± 4.54 ^aA^	86.17 ± 6.10 ^aA^
7	Caffeic acid derivative (CAD)									
	Early (I)	0.0	0.0	0.0	0.30 ± 0.11 ^cB^	8.67 ± 1.92 ^aA^	0.0	0.0	3.13 ± 0.12 ^bB^	9.34 ± 0.96 ^aB^
	Middle (II)	0.0	0.0	0.0	14.73 ± 5.92 ^bA^	2.51 ± 1.44 ^cB^	0.0	0.0	27.31 ± 0.59 ^aA^	17.92 ± 1.89 ^bA^
8	*p*-Coumaric acid derivative (*p*-CAD1)									
	Early (I)	0.0	0.0	0.0	Tr	Tr	0.0	0.0	0.0	0.0
	Middle (II)	0.0	0.0	0.0	0.59 ± 0.30 ^c^	0.10 ± 0.00 ^d^	0.0	0.0	5.88 ± 0.30 ^a^	1.13 ± 0.19 ^b^
9	Caffeic acid acetilglycoside (CAAG)									
	Early (I)	0.0	0.0	0.0	Tr	Tr	0.0	0.0	3.16 ± 0.66 ^abB^	4.46 ± 1.05 ^aB^
	Middle (II)	0.0	0.0	0.0	0.28 ± 0.08 ^b^	0.11 ± 0.01 ^b^	0.0	0.0	13.81 ± 1.12 ^aA^	13.06 ± 2.37 ^aA^
10	*p*-Coumaric acid (*p*-CA)									
	Early (I)	Tr	Tr	Tr	12.87 ± 1.13 ^bB^	23.39 ± 3.50 ^aA^	Tr	Tr	0.63 ± 0.10 ^cB^	0.09 ± 0.00 ^dB^
	Middle (II)	Tr	Tr	Tr	27.51 ± 4.14 ^aA^	19.94 ± 0.11 ^abAB^	Tr	Tr	25.46 ± 1.80 ^aA^	21.26 ± 3.75 ^aA^
11	*p*-Coumaric acid acetylglycoside (*p*-CAAG)									
	Early (I)	0.0	0.0	Tr	0.0	Tr	0.0	0.0	Tr	Tr
	Middle (II)	0.0	0.0	0.0	5.13 ± 1.50 ^b^	1.67 ± 0.77 ^c^	0.0	0.0	7.29 ± 0.51 ^a^	5.59 ± 0.37 ^b^
12	*p*-Coumaric acid derivative (*p*-CAD2)									
	Early (I)	0.0	0.0	0.0	105.38 ± 10.93 ^abB^	125.31 ± 22.26 ^aB^	0.0	0.0	56.00 ± 3.79 ^cB^	36.22 ± 145 ^dB^
	Middle (II)	0.0	0.0	0.0	188.02 ± 22.21 ^bA^	181.98 ± 15.08 ^bA^	0.0	0.0	262.10 ± 3.26 ^aA^	184.75 ± 19.59 ^bA^
13	*trans*-Ferulic acid (*t*-FA)									
	Early (I)	0.0	Tr	Tr	1.57 ± 0.54 ^bB^	9.01 ± 2.33 ^aA^	0.0	0.0	Tr	Tr
	Middle (II)	0.0	Tr	Tr	5.24 ± 1.73 ^bA^	9.49 ± 2.35 ^aA^	0.0	0.0	3.90 ± 0.31 ^b^	Tr
14	*p*-Coumaric acid derivative (*p*-CAD3)									
	Early (I)	0.0	0.0	0.0	Tr	Tr	0.0	0.0	3.32 ± 0.57 ^aB^	3.01 ± 0.41 ^aB^
	Middle (II)	0.0	0.0	0.0	1.51 ± 0.00 ^d^	1.91 ± 1.00 ^c^	0.0	0.0	30.01 ± 0.44 ^aA^	5.43 ± 1.45 ^bAB^
	Total phenolic compounds									
	Early (I)	102.73 ± 1.86 ^dA^	85.77 ± 13.34 ^eA^	71.23 ± 2.09 ^efA^	243.46 ± 20.80 ^aB^	267.52 ± 28.01 ^aB^	107.88 ± 0.71 ^dA^	82.45 ± 5.67 ^eA^	172.22 ± 9.58 ^bB^	126.69 ± 9.66 ^cB^
	Middle (II)	56.18 ± 14.13 ^eB^	68.66 ± 11.96 ^deA^	88.62 ± 1.09 ^cA^	454.62 ± 67.72 ^abA^	381.97 ± 27.32 ^bA^	73.01 ± 1.18 ^dB^	74.54 ± 11.81 ^dA^	515.76 ± 9.44 ^aA^	392.72 ± 4.21 ^bA^

The values in each row are the mean of six independent determinations (six avocados) ± standard deviation (n = 6). Different lowercase superscript letters in the same row indicate significant differences. Letters in different uppercase superscripts in the same column per compound indicate significant difference (*p* < 0.05) by the Tukey test. Tr = traces. (*) corresponds to ready to eat fruit (edible ripeness) subjected to ripening at shelf-life conditions (20 °C) after 30 and 50 days of controlled atmosphere or hydrothermal treatment followed by controlled atmosphere storage.

## Data Availability

Not applicable.

## Supplementary Materials

The following are available online at https://www.mdpi.com/article/10.3390/plants10112427/s1, Figure S1: Biplot displaying the samples and variables overlaid for the whole dataset (early and middle harvest fruit), Figure S2: Biplot displaying the samples and variables overlaid for the early harvest dataset, Figure S3: Biplot displaying the samples and variables overlaid for middle harvest dataset.
