# Supplementary material for: The Effect of Hydrothermal Treatment on Metabolite Composition of Hass Avocados Stored in a Controlled Atmosphere
Source: Plants (Basel). 2021 Nov 10;10(11):2427. doi: 10.3390/plants10112427 (PMC8626034; doi:10.3390/plants10112427)
Supplement: Supplementary file 1 [file plants-10-02427-s001.zip › plants-1418264-SI.pdf]

Figure S1

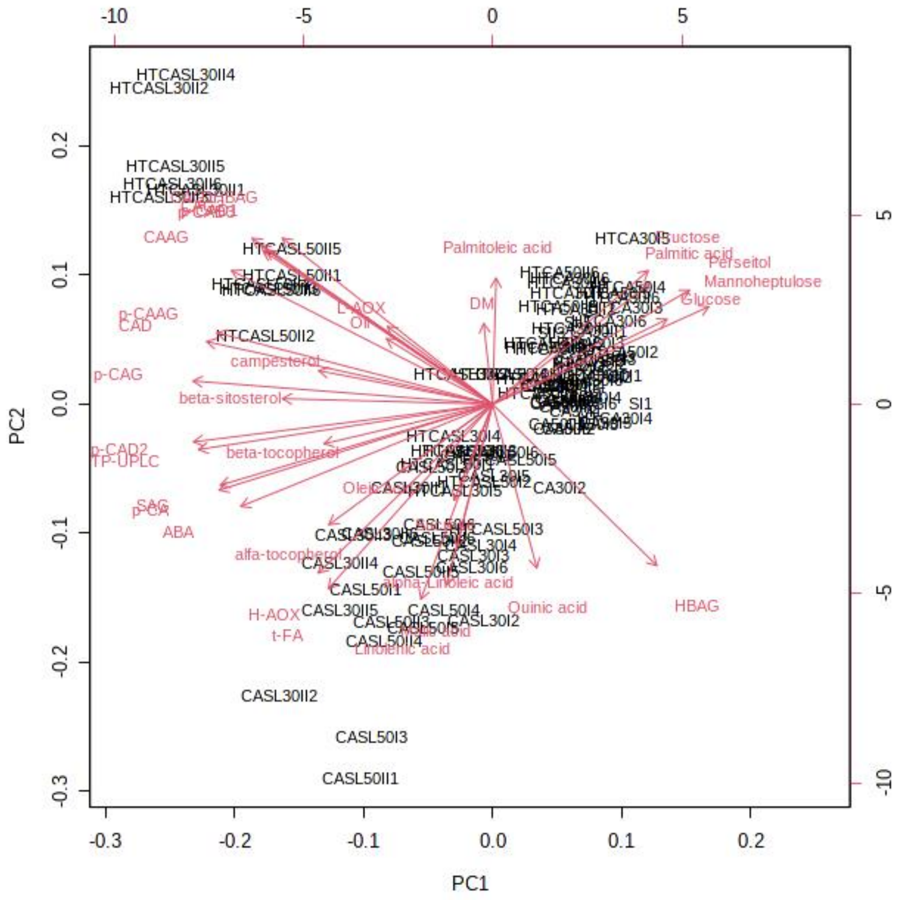

Biplot displaying the samples and variables overlaid for the whole dataset (early and middle harvest fruit). CAI/HTCAI = Initial day early harvest; CAII/HTCAII = Initial day middle harvest; CA30I and CA50I = controlled atmosphere for 30 and 50 d early harvest; CA30II and CA50II = controlled atmosphere for 30 and 50 d middle harvest; CASL30I and CASL50I = controlled atmosphere for 30 and 50 d early harvest after shelf life period; CASL30II and CASL50II = controlled atmosphere for 30 and 50 d middle harvest after shelf life period; HTCA30I and HTCA50I = hydrothermal treatment for 30 and 50 d early harvest; HTCA30II and HTCA50II = hydrothermal treatment for 30 and 50 d middle harvest; HTCASL30I and HTCASL50I = hydrothermal treatment for 30 and 50 d early harvest after shelf life period; HTCASL30II and HTCASL50II = hydrothermal treatment for 30 and 50 d middle harvest after shelf life (numbers from 1 to 6 represent the replicates). DM = dry matter, ABA= abscisic acid, H-AOX= hydrophilic antioxidant activity, L-AOX= lipophilic antioxidant activity, DHBAG = dihydroxybenzoic acid glycoside, SAG = syringic acid glycoside, CAG1 = caffeic acid glycoside 1, CAG2 = caffeic acid glycoside 2, HBAG = hydroxybenzoic acid glycoside, *p*-CAG = *p*-coumaric acid glycoside, CAD= caffeic acid derivative, *p*-CAD1 = *p*-coumaric acid derivative, CAAG = caffeic acid acetilglycoside, *p*-CA = *p*-coumaric acid, *p*-CAAG = *p*-coumaric acid acetylglycoside, *p*-CAD2 = *p*-coumaric acid derivative, *t*-FA = *trans*-ferulic acid, *p*-CAD3 = *p*-coumaric acid derivative.

Figure S2

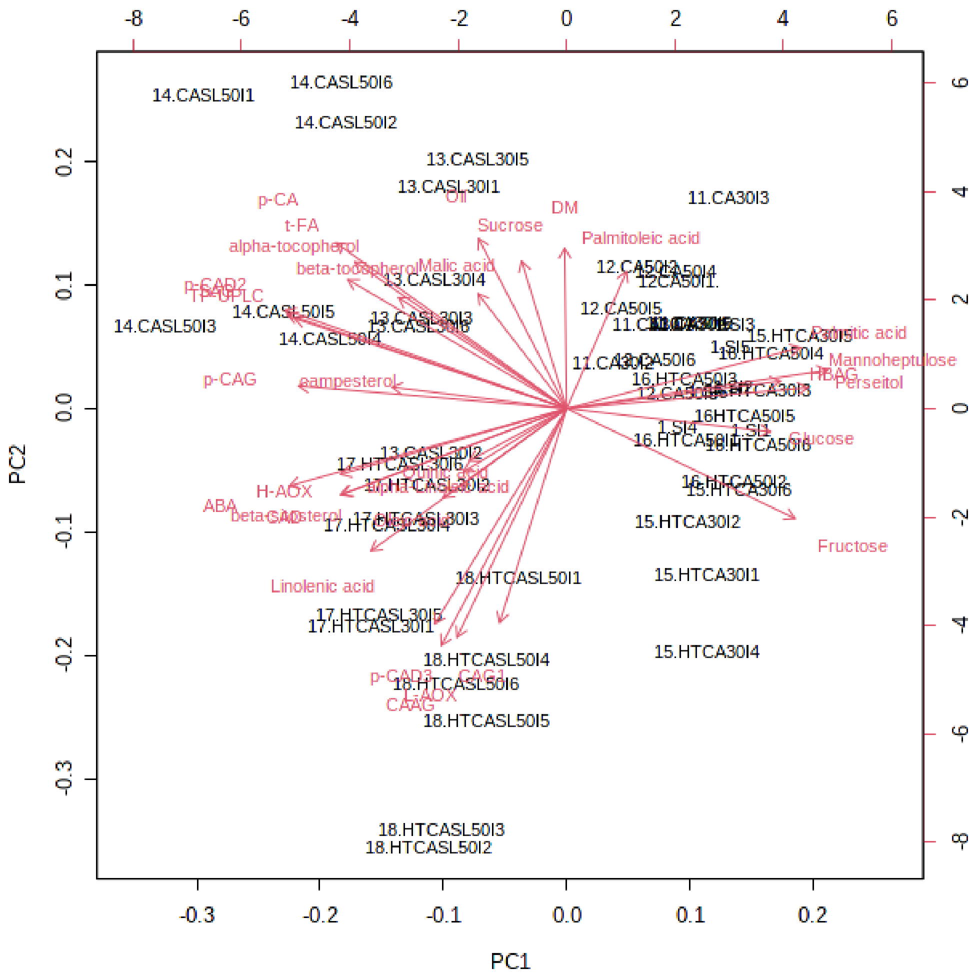

Biplot displaying the samples and variables overlaid for the early harvest dataset. CAI/HTCAI = Initial day early harvest; CA30I and CA50I = controlled atmosphere for 30 and 50 d early harvest; CASL30I and CASL50I = controlled atmosphere for 30 and 50 d early harvest after shelf life period; HTCA30I and HTCA50I = hydrothermal treatment for 30 and 50 d early harvest; HTCASL30I and HTCASL50I = hydrothermal treatment for 30 and 50 d early harvest after shelf life (numbers from 1 to 6 represent the replicates). DM = dry matter, ABA= abscisic acid, H-AOX= hydrophilic antioxidant activity, L-AOX= lipophilic antioxidant activity, DHBAG = dihydroxybenzoic acid glycoside, SAG = syringic acid glycoside, CAG1 = caffeic acid glycoside 1, CAG2 = caffeic acid glycoside 2, HBAG = hydroxybenzoic acid glycoside, *p*-CAG = *p*-Coumaric acid glycoside, CAD= caffeic acid derivative, *p*-CAD1 = *p*-coumaric acid derivative, CAAG = caffeic acid acetilglycoside, *p*-CA = *p*-coumaric acid, *p*-CAAG = *p*-coumaric acid acetylglycoside, *p*-CAD2 = *p*-coumaric acid derivative, *t*-FA = *trans*-ferulic acid, *p*-CAD3 = *p*-coumaric acid derivative.

Figure S3

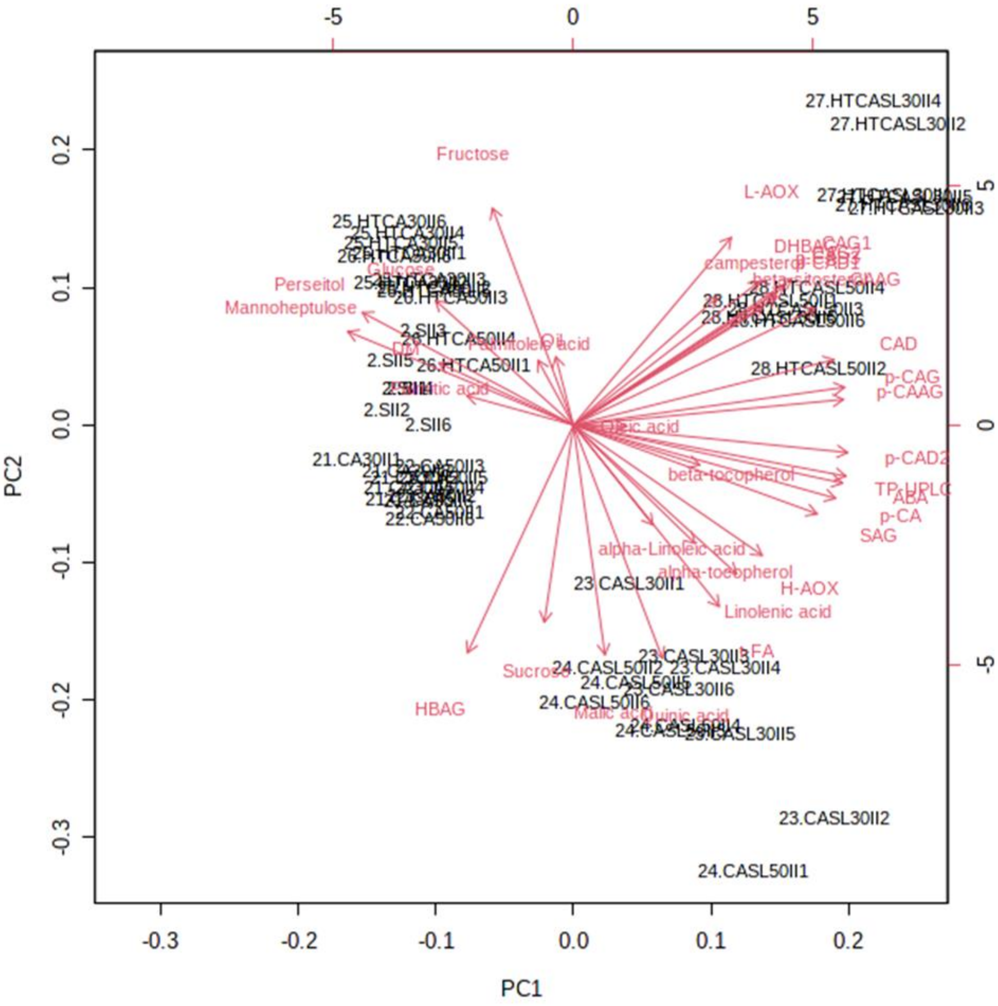

Biplot displaying the samples and variables overlaid for middle harvest dataset. CAII/HTCAII= Initial day middle harvest; CA30II and CA50II = controlled atmosphere for 30 and 50 d middle harvest; CASL30II and CASL50II = controlled atmosphere for 30 and 50 d middle harvest after shelf life period; HTCA30II and HTCA50II = hydrothermal treatment for 30 and 50 middle harvest; HTCASL30II and HTCASL50II = hydrothermal treatment for 30 and 50 d middle harvest after shelf life period (numbers from 1 to 6 represent the replicates). DM = dry matter, ABA= abscisic acid, H-AOX= hydrophilic antioxidant activity, L-AOX= lipophilic antioxidant activity, DHBAG = dihydroxybenzoic acid glycoside, SAG = syringic acid glycoside, CAG1 = caffeic acid glycoside 1, CAG2 = caffeic acid glycoside 2, HBAG = hydroxybenzoic acid glycoside, *p*-CAG = *p*-coumaric acid glycoside, CAD= caffeic acid derivative, *p*-CAD1 = *p*-coumaric acid derivative, CAAG = caffeic acid acetilglycoside, *p*-CA = *p*-coumaric acid, *p*-CAAG = *p*-coumaric acid acetylglycoside, *p*-CAD2 = *p*-coumaric acid derivative, *t*-FA = *trans*-ferulic acid, *p*-CAD3 = *p*-coumaric acid derivative.
